# A *Drosophila* screen identifies a role for histone methylation in ER stress preconditioning

**DOI:** 10.1093/g3journal/jkad265

**Published:** 2023-12-14

**Authors:** Katie G Owings, Clement Y Chow

**Affiliations:** Department of Human Genetics, University of Utah School of Medicine, EIHG 5200, 15 North 2030 East, Salt Lake City, UT 84112, USA; Department of Human Genetics, University of Utah School of Medicine, EIHG 5200, 15 North 2030 East, Salt Lake City, UT 84112, USA

**Keywords:** stress preconditioning, *Drosophila melanogaster*, DGRP, ER stress, heat shock, natural genetic variation

## Abstract

Stress preconditioning occurs when transient, sublethal stress events impact an organism's ability to counter future stresses. Although preconditioning effects are often noted in the literature, very little is known about the underlying mechanisms. To model preconditioning, we exposed a panel of genetically diverse *Drosophila melanogaster* to a sublethal heat shock and measured how well the flies survived subsequent exposure to endoplasmic reticulum (ER) stress. The impact of preconditioning varied with genetic background, ranging from dying half as fast to 4 and a half times faster with preconditioning compared to no preconditioning. Subsequent association and transcriptional analyses revealed that histone methylation, and transcriptional regulation are both candidate preconditioning modifier pathways. Strikingly, almost all subunits (7/8) in the Set1/COMPASS complex were identified as candidate modifiers of preconditioning. Functional analysis of *Set1* knockdown flies demonstrated that loss of *Set1* led to the transcriptional dysregulation of canonical ER stress genes during preconditioning. Based on these analyses, we propose a preconditioning model in which Set1 helps to establish an interim transcriptional “memory” of previous stress events, resulting in a preconditioned response to subsequent stress.

## Introduction

Organisms routinely face many stressors, including changes in temperature, viral infection, exposure to environmental toxins, hypoxia, and ischemia (Hoffmann and Hercus 2000). Understanding how an individual can respond to numerous insults over a lifetime is an ongoing challenge. In laboratory environments, naïve cells and organisms are commonly used to dissect the mechanisms underlying stress response pathways. However, this does not reflect the complex history of stresses that would naturally occur.

Efficient stress responses are vital for producing and maintaining a healthy proteome (Chaudhuri and Paul 2006; Rendleman *et al.* 2018). Therefore, cells have many canonical stress response pathways that combat cellular stresses. The endoplasmic reticulum (ER) is responsible for folding approximately 30% of all polypeptides, which is an error-prone process disrupted by many stressors (Guerriero and Brodsky 2012; Hetz 2012). The ER stress pathway is one of the most thoroughly characterized canonical stress response pathways. Three ER membrane sensors, IRE1, ATF6, and PERK, detect misfolded proteins and respond by initiating the unfolded protein response (UPR). The UPR includes a robust transcriptional cascade that upregulates genes whose protein products refold or degrade misfolded proteins (Hetz 2012). If the cell cannot achieve ER homeostasis, then apoptosis occurs. An effective ER stress response is critical for healthy development and aging. Improper proteostasis and a decline in the ER stress response contribute to many diseases, such as Alzheimer's disease, Parkinson's disease, Type 2 diabetes, and more (Chaudhuri and Paul 2006; Back and Kaufman 2012; Brown and Naidoo 2012; Cnop *et al.* 2012; Labbadia and Morimoto 2015). An essential step in understanding how the ER stress response impacts disease is understanding how the stress response varies with genetic background and previous stress.

Natural genetic variation is a powerful tool for investigating canonical stress response pathways. Incorporating genetic variation into ER stress has revealed new genes and pathways involved in the ER stress response (Russell and Chow 2022; Chow *et al.* 2013, 2015, 2016; Palu and Chow 2018; Palu *et al.* 2019). Much of what we understand about the ER stress response comes from studies that examine this response in isolation, using a single genetic background. In reality, ER stress occurs in a complex milieu of previous stresses that likely impact how the cell responds, and this likely varies with genetic background.

Preconditioning is a long-observed example of an organism's ability to adapt to numerous assaults, whereby transient exposure to stress affects the organism's ability to respond to subsequent stresses (Hayashi *et al.* 2003; Hung *et al.* 2003; Fouillet *et al.* 2012; Kennedy *et al.* 2014). There are many documented cases of preconditioning modifying the outcome of canonical stress pathways. For example, renal epithelial cells preconditioned with various pharmaceutical inducers of ER stress are resistant to subsequent peroxide-induced cell death (Hung *et al.* 2003). Exposing *Caenorhabditis elegans* to mitochondrial stress during larval development increases their ability to respond to and recover from heat stress as adults (Labbadia *et al.* 2017). In a mouse model of Parkinson's disease, preconditioning mice with ER stress through tunicamycin injections is neuroprotective from subsequent 6-OHDA injections (Fouillet *et al.* 2012). While most of these are examples of positive effects, preconditioning can also have negative effects. For example, in cultured mammalian neurons, proteasomal inhibition can negatively impact stress granule formation and further responses to other stresses (Shelkovnikova *et al.* 2017). Extrinsic factors such as age and diet can also result a negative preconditioning effect (Pomatto and Davies 2017; Holcombe and Weavers 2022). Although this long-observed phenomenon has been documented, the molecular mechanisms are unknown, and stress response pathways continue to be studied in isolation.

We used natural genetic variation to understand the impact of stress preconditioning on the ER stress response. Here, we report the results of a stress preconditioning screen performed using a genetically diverse panel of *Drosophila melanogaster*. The screen revealed that preconditioning outcomes are strongly dependent on genetic background. We identified candidate modifier genes from association analyses of the preconditioning screen and identified differentially expressed genes between strains with opposing preconditioning outcomes using transcriptional analyses. Together, these analyses revealed that immunity, transcriptional regulation, and histone methylation might play a role in the underlying mechanisms of preconditioning. Strikingly, we identified nearly all the components of the Set1/COMPASS complex as candidate modifiers. We investigated the impact of the loss of *Set1*, a conserved histone H3 lysine 4 (H3K4) methyltransferase in the Set1/COMPASS complex, on preconditioning. We found that *Set1* knockdown modifies preconditioning outcomes and leads to the transcriptional dysregulation of a subset of genes during preconditioning. We posit that after initial stress, Set1 plays a critical role in creating a transient transcriptional “memory” of the event, resulting in a preconditioned response to subsequent stress.

## Materials and methods

### *Drosophila* lines and maintenance

Flies were maintained at 25°C on a standard diet based on the Bloomington Stock Center standard medium with malt and without soy flour. Flies were on a 12-h light/dark cycle. For the stress preconditioning screen, DGRP strains were obtained from the Bloomington *Drosophila* Stock Center. For the *Set1* functional work, a *Tubulin-*GAL4 driver (Bloomington *Drosophila* Stock Center: 5138), Attp40 (36304), and Set1 RNAi (40931) were used.

### Stress preconditioning assay

One hundred and seventy-seven strains from the DGRP were used for the stress preconditioning screen. For each strain, 200 males were collected and placed in 10 vials of 20 males each. Each male was between 2–8 days old and had recovered a minimum of 2 days since their last exposure to CO_2_ when they were exposed to stress. Males were chosen to avoid interaction between stress responses and adult survival of mated vs unmated females. For preconditioning, 5 vials of 20 flies (100 flies total) were heat shocked by placing them into empty vials and submerging in a 35 ± 1°C water bath for 30 min. All heat shocks were performed in the morning, between 9 Am and 12 Pm. All flies were placed back on standard media and allowed to recover at 25°C for 4 h. TM food consisted of 8 µM TM (Sigma-Aldrich CAS Number: 11089–65-9) dissolved in DMSO (Millipore Sigma CAS Number: 67-68-5), 1.3% agarose (BioRad #1620102), and 1% sucrose (Millipore Sigma CAS Number: 57-50-1) in DI water (similar to previous studies; Chow *et al.* 2013). After recovery, all flies were transferred into vials containing 5 ml of tunicamycin (TM) food to induce ER stress. TM inhibits N-linked glycosylation, leading to ER stress, and is commonly utilized in ER stress studies (Heifetz *et al.* 1979; Oslowski and Urano 2011; Chow *et al.* 2013; Ryoo 2015; Dong *et al.* 2018). Flies were monitored every 2 h during the light cycle (8 AM–8 PM MST) and the number of dead flies were recorded. The control, no preconditioning flies, were treated in the same manner, but were not exposed to heat shock.

The *Set1* KD stress preconditioning assay used the same protocol. The only adjustment is that flies were monitored every 2 h between 8 AM–12 AM MST (additional 4 h during the dark cycle) once death was observed. Each replicate is made up of 100 *Set1* KD and 100 control flies that were collected, subjected to the stress preconditioning screen, and monitored for survival at the same time.

### Hazard ratios

Survival analysis was performed using the Survival package in R (R version 4.2.0; survival package version 3.3-1; running under Windows 10 ×64) and detailed code is located at https://github.com/kgowings/ER_stress_preconditioning/blob/main/R_plots%26stats/HR_and_BarPlot.R. The coxph test was performed to calculate the Cox proportional hazards ratio (HR) for each strain. The HR compares the death rate of the preconditioning group to the death rate of the control group within each strain. The HR takes into account all 100 flies exposed to heat stress and then ER stress and all 100 control flies exposed to only ER stress.

### Genome-wide association study

Genome-wide association (GWA) was performed as previously described (Chow *et al.* 2016). DGRP genotype files were downloaded from the website: http://dgrp2.gnets.ncsu.edu/data.html. The HR calculated from the preconditioning screen for 177 DGRP lines was regressed on each SNP. GEMMA (v. 0.94) (Zhou and Stephens 2012) was used to estimate a centered genetic relatedness matrix and perform association tests using the following linear mixed model:


$$\begin{array}{rl} & y=\alpha +x\beta +u+\varepsilon \\ & u∼\mathrm{MV}N_{n}(0,\lambda \tau^{-1}K)\\ & \varepsilon ∼\mathrm{MV}N_{n}(0,\tau^{-1}I_{n}), \end{array}$$

where y is the n-vector of the HR for the n lines, α is the intercept, x is the n-vector of marker genotypes, β is the effect size of the marker. u is an n × n matrix of random effects with a multivariate normal distribution (MVNn) that depends on λ, the ratio between the 2 variance components, τ^−1^, the variance of residuals errors, and where the covariance matrix is informed by K, the calculated n × n marker-based relatedness matrix. K accounts for all pairwise nonrandom sharing of genetic material among lines. ɛ, is an n-vector of residual errors, with a multivariate normal distribution that depends on τ^−1^ and I_n_, the identity matrix. Full GWA output located at https://figshare.com/articles/dataset/StressPreconditioningDGRP_GWAoutput_txt/22266238. When evaluating candidate polymorphisms, we used the significance output “P_score.” Variants were filtered for MAF (≥0.05) and nonbiallelic sites were removed.

SNPs were assigned to genes within ±1 kb using the variant annotation file based on FB5.57 (dgrp.fb557.annot.txt) from the DGRP website, http://dgrp2.gnets.ncsu.edu/data.html. If multiple genes are within ±1 kb from a given SNP, the SNP was assigned to a single gene by prioritizing the variant type as follows: exon > UTR > intron > upstream or downstream. Human orthologues for each fly gene were chosen based on the greatest DIOPT score (Hu *et al.* 2011), with a minimum DIOPT score of 5. All of the GWAS code used to perform this analysis and prepare the output for GSEA is detailed at https://github.com/kgowings/ER_stress_preconditioning/tree/main/GWA%26GSEA and GEMMA documentation can be found at https://github.com/genetics-statistics/GEMMA.

### Gene set enrichment analysis

Gene set enrichment analysis (GSEA) analysis was performed as previously described (Subramanian *et al.* 2005; Palu *et al.* 2019; Talsness *et al.* 2020). All polymorphisms from the stress preconditioning GWA (Supplementary File 1) were assigned to a gene as described in the above section. Genes were organized into a rank-list based on their enrichment for polymorphisms, and genes were assigned to GO categories. GSEA determines whether the top of the newly generated rank-list is enriched in genes belonging to any GO categories or if the genes in the category are randomly distributed throughout the list. Calculation of enrichment score was performed as described by Subramanian *et al.* (2005) (for code see Figshare: https://doi.org/10.25387/g3.9808379). Only GO categories with a corrected *P*-value ≤ 0.05, number of genes ≥ 5, and an enrichment score ≥ 0.50 were considered.

### RNAseq

mRNA sequencing was performed on total RNA from whole male 2-day old flies (10 flies per group sample). DGRP lines RAL69, RAL93, RAL359, RAL387, and RAL409 made up the beneficial group and RAL195, RAL304, RAL335, RAL737, and RAL819 made up the detrimental group. Flies were given 48 h to recover from their last CO_2_ exposure. The no treatment flies were frozen down at the same time as the heat shock samples. The heat shock samples from each line were heat shocked for 30 min at 35 ± 1°C and then frozen down immediately postheat shock.

mRNA sequencing was performed on 20 samples (10 genotypes × 2 treatments × 1 replicate). RNA was extracted using a Direct-zol RNA Miniprep (Zymo Research R2061) using TRIzol Reagent (ThermoFisher Cat # 15596026) and including the DNAse step. Samples were prepared and sequenced by the Huntsman Cancer Institute High-Throughput Genomics Core. The 20 samples were sequenced on the NovaSeq 50 × 50 bp Sequencing, for a total of approximately 25 million paired reads per sample. Fastq files were trimmed using seqtk v1.2 software (for FastQ and processed files see GEO repository: GSE226958). RNAseq reads were aligned to the *D. melanogaster* reference genome (assembly BDGP6.28, Ensembl release 102) using Bowtie2 v2.2.9 software (Langmead and Salzberg 2012), and alignment files were sorted and converted using Samtools v1.12 (Li *et al.* 2009).

Read counts were normalized using the default normalization method in DESeq2 (Love *et al.* 2014) package in R. Principle components analysis (PCA) was performed to identify outliers (Supplementary Fig. 3). RAL409 was identified as an outlier and was removed for further analyses. Outliers were detected by calculating the PC1, PC2, and distance from the center for each sample. Then we calculated the mean +2 times the standard deviation for each and set them as the threshold for outliers. Any samples that exceded any of the 3 thresholds was counted as an outlier and removed for further analysis. The remaining samples were reanalyzed using Deseq2 v1.28.1. Differentially expressed genes in the beneficial group (compared to detrimental group) were identified before treatment and immediately postheat shock. Remaining samples were renormalized and assessed using linear models with the DESeq2 package. Genes were considered significantly differentially expressed if the adjusted *P*-value ≤ 0.10. We chose this less stringent *P*-value threshold due to the noise introduced by using 5 different DGRP strains as biological replicates. In this preliminary characterization of our preconditioning model, we wanted readers to see a broader report of the RNAseq results that are not too restricted by *P*-value cutoffs since all *P*-values are displayed. All of our RNAseq code is available at https://github.com/kgowings/ER_stress_preconditioning/tree/main/RNAseq_DESeq.

### RT-qPCR

Each sample contained 12 adult male flies that were 4–7 days old and had been off CO_2_ for 3 days when exposed to stress. Thirty samples were collected (5 timepoints × 2 genotypes × 3 replicates), and RNA was extracted using a Direct-zol RNA Miniprep (Zymo Research R2061) using TRIzol Reagent (ThermoFisher Cat # 15596026) and including the DNAse step. RNA was converted to cDNA using a ProtoScript® II First Strand cDNA Synthesis Kit (NEB Cat # E6560L). RT-qPCR was performed using a QuantStudio 3 96-well 0.2 ml block instrument and PowerUp SYBR Green Master Mix (ThermoFisher Cat # A25741). We used primers from the FlyPrimerBank (Hu *et al.* 2013) located at http://www.flyrnai.org/flyprimerbank: *Set1* (PP4079), *Hsp70* (PD40143), *Hsp26* (PD70434), *Hsp83* (PD70430), *GstD2* (PP14716), *Ugt37A3* (PP30182), *Sil1* (PP30411), and *RpL19* (PP10148).

Since these were previously established primers, primer efficiency was not calculated using standard curves. Results were analyzed using the Delta-Delta Ct method. Gene expression was normalized to the expression of a housekeeping gene, *RpL19*. Then, the normalized gene expression at each timepoint was normalized to the “No Treatment” timepoint.

## Results

### Genetic background modifies preconditioning outcomes

**Fig. 1.**
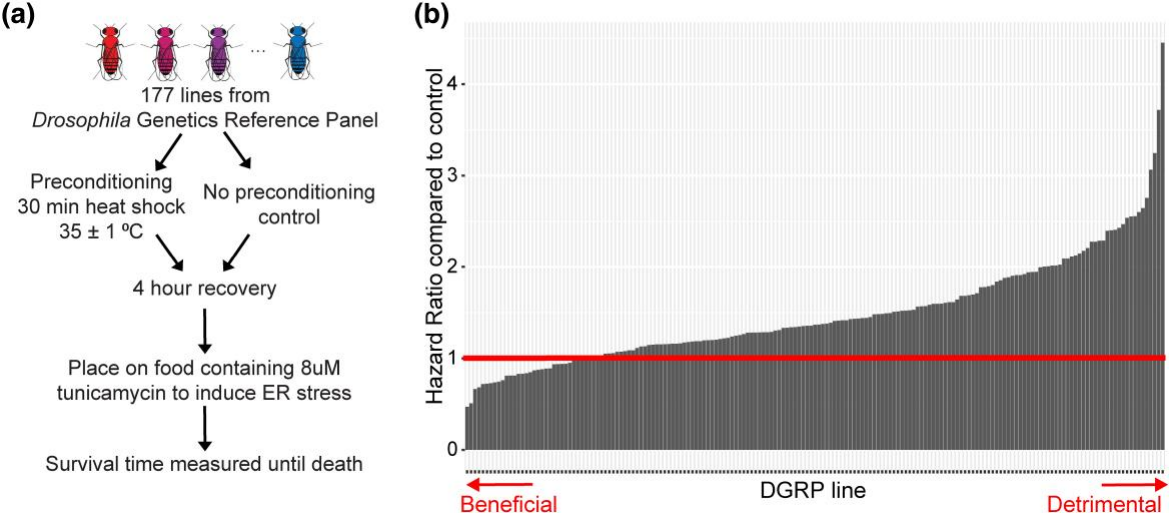
Genetic background alters the effect of preconditioning on ER stress survival times. a) Experimental design of the stress preconditioning screen performed on 177 lines of the *Drosophila* Genetics Reference Panel (DGRP). b) Results of stress preconditioning screen. Each bar represents a different DGRP strain. Cox proportional hazard ratio: [rate of death on ER stress with preconditioning]/[rate of death on ER stress without preconditioning]. A hazard ratio < 1 indicates preconditioning had a beneficial effect on ER stress survival, a hazard ratio = 1 indicates no effect, and a hazard ratio > 1 indicates a detrimental effect. The horizontal line marks this transition point at hazard ratio = 1.

We subjected 177 strains of the *Drosophila* Genetic Reference Panel (DGRP) to a stress preconditioning screen to assess the impact of genetic variation on how preconditioning affects the ER stress response. The DGRP, a collection of fully sequenced, inbred *Drosophila* strains derived from a natural population, is a powerful tool for uncovering novel effects of genotypic background on biological processes (Mackay *et al.* 2012). For our preconditioning screen, we exposed males of each DGRP strain to heat stress preconditioning (or no preconditioning control), allowed them to recover for 4 h, placed them on food containing tunicamycin (TM) to induce ER stress, and monitored survival (Fig. 1a).

A number of unique stress combinations have been used to model preconditioning, including multiple exposures to a single stress (Hercus *et al.* 2003; Ding *et al.* 2012) or the application of 2 different stresses (Hayashi *et al.* 2003; Hung *et al.* 2003; Kennedy *et al.* 2014; D’Urso *et al.* 2016; Labbadia *et al.* 2017). We chose to use 2 mostly independent stressors that required unique stress response genes, rather than repeating a single stress twice (Chow *et al.* 2015; Humburg *et al.* 2016). This reduces the likelihood that the effect of preconditioning is only due to proteins or transcripts generated during the initial stress and are still present during the secondary stress. Sublethal heat stress was chosen as the preconditioning stress because it is applied to flies uniformly, causes a robust response, has been extensively characterized, and begins within minutes of heat application (Ashburner and Bonner 1979; Humburg *et al.* 2016; Mahat *et al.* 2016). TM induces ER stress by inhibiting N-linked glycosylation and is commonly utilized in ER stress studies (Heifetz *et al.* 1979; Oslowski and Urano 2011; Chow *et al.* 2013; Ryoo 2015; Dong *et al.* 2018). TM-induced ER stress ultimately leads to the death of all flies (Chow *et al.* 2013). Therefore, the phenotypic outcome measured in this screen was the survival time under TM-induced ER stress.

We compared survival curves of preconditioned and control flies from each DGRP strain to determine how preconditioning affects each strain's response to ER stress. Survival was analyzed with the Cox proportional hazards model to generate a hazard ratio for each strain that compares the death rate on ER stress with and without heat stress preconditioning (Abd Elhafeez *et al.* 2021) (Supplementary File 1). A hazard ratio value of 1 indicates no significant change due to preconditioning, <1 indicates slower death in the preconditioned group compared to control (beneficial), and >1 indicates faster death in the preconditioned group (detrimental).

The stress preconditioning screen revealed that the effect of preconditioning on the response to ER stress is dependent on genetic background (Fig. 1b). The impact of preconditioning on ER stress-induced death rates ranges from dying half as fast (hazard ratio = 0.47, *P* = 2.36 × 10^−7^) to 4.5 times faster (hazard ratio = 4.45, *P* < 2.0 × 10^−16^) compared to no preconditioning. Since this experiment examines preconditioning in a single generation of the DGRP, we are unable to comment on the heritability of preconditioning effects. To address this limitation, future studies would have to include parent–offspring comparisons of stress preconditioning effects to definitively address the heritability of this trait. There is no correlation in the DGRP between responses to preconditioning and previously reported ER stress responses (*r* = −0.16; *P* = 0.13) (Chow *et al.* 2013), heat tolerance (*r* = −0.047; *P* = 0.67) (Lecheta *et al.* 2020), or longevity (*r* = 0.036; *P* = 0.65) (Huang *et al.* 2020), indicating that these individual factors do not drive preconditioning outcomes (Supplementary Fig. 2). Therefore, the variability in preconditioning outcomes directly results from unique, underlying genetic variation in the DGRP and is not simply the sum of the effects of variation on stress tolerance in general and overall longevity.

### GWA analysis identifies candidate modifier genes of preconditioning

We performed a GWA analysis using the hazard ratios generated for each DGRP strain to identify candidate modifier genes underlying the variable preconditioning outcomes. We applied a linear mixed model to query 1,885,860 polymorphisms (MAF ≥ 0.05) to identify variants that are significantly associated with outcomes from our preconditioning screen (results located at: https://figshare.com/articles/dataset/StressPreconditioningDGRP_GWAoutput_txt/22266238). The quantile–quantile (qq) plot demonstrated an appropriate fit to the linear mixed model, but with less enrichment than we expected (Supplementary Fig. 1).

Evaluating the role of any specific SNP is difficult due to limitations imposed by multiple testing. Therefore, in this study, we put little emphasis on individual polymorphisms. Instead, we prioritized identifying potential modifier genes, which has been a very successful approach in previous DGRP screens (Chow *et al.* 2013, 2016; Palu *et al.* 2019; Talsness *et al.* 2020). If a SNP fell within an annotated gene, we assigned it to that gene. If a SNP was in an intergenic region, we assigned it to the closest gene within 1 kb. We did not evaluate SNPs that were more than 1 kb from the closest gene, so our analysis excludes variants that lie outside this range and may have transregulatory effects.

With a standard *P*-value cutoff of *P* ≤ 1 × 10^−5^ used in most DGRP studies (Chow *et al.* 2013, 2016; Palu *et al.* 2019; Talsness *et al.* 2020), the GWA identified only 6 polymorphisms associated with stress preconditioning outcomes. Of the 6 polymorphisms, 5 were in a known gene: 1 in a 3′ UTR, 3 in introns, and 1 synonymous variant in a protein-coding exon. These variants are the top 5 variants displayed in Table 1. Of these 5 *Drosophila* genes, 2 have known human orthologs, *Pdp1* (human ortholog: *HLF*) and *CG44837* (*DPEP3*). *Pdp1* (*HLF*) is particularly interesting because it is the top candidate modifier with a human orthologue. *Pdp1* (*HLF*) is a widely expressed transcription factor critical for proper development and circadian rhythm (Lin *et al.* 1997; Reddy *et al.* 2000).

**Table 1.** Candidate modifier genes identified from GWA.

Rank order	Gene	FBgn	Human ortholog	*P*-value
1	*CG32204*	FBgn0052204	*—*	1.40E−06
2	*Pdp1*	FBgn0016694	*HLF*	4.97E−06
3	*TwdlJ*	FBgn0039440	*—*	5.13E−06
4	*CG44837*	FBgn0266100	*DPEP3*	6.80E−06
5	*CG6024*	FBgn0036202	*—*	7.69E−06
6	*cep290*	FBgn0035168	*CEP290*	1.27E−05
7	*CR45184*	FBgn0266694	*—*	1.53E−05
8	*LpR2*	FBgn0051092	*VLDLR*	1.57E−05
9	*Ckn*	FBgn0033987	*—*	1.73E−05
10	*Sf3b3*	FBgn0035162	*SF3B3*	2.67E−05
11	*Hdc*	FBgn0010113	*HECA*	2.83E−05
12	*PGAP1*	FBgn0029789	*PGAP1*	2.91E−05
13	*Nmo*	FBgn0011817	*NLK*	3.42E−05
14	*Tpl94D*	FBgn0051281	*—*	4.36E−05
15	*Wnt10*	FBgn0031903	*WNT10B*	4.56E−05
16	*Ser7*	FBgn0019929	*—*	4.64E−05
17	*CG17197*	FBgn0039367	*ZDHHC24*	4.70E−05
18	*Fry*	FBgn0016081	*FRYL*	5.21E−05
19	*CR44971*	FBgn0266306	*—*	5.30E−05
20	*CG17097*	FBgn0265264	*LIPA/F/K/M/N*	5.37E−05
21	*CG11880*	FBgn0039637	*SLC44A2/4*	5.45E−05
22	*CG34375*	FBgn0085404	*—*	5.89E−05
23	*Side*	FBgn0016061	*—*	6.03E−05
24	*TyrR*	FBgn0038542	*—*	6.15E−05
25	*CG12206*	FBgn0029662	*GRXCR1*	6.20E−05
26	*Hs6st*	FBgn0038755	*HS6ST1*	6.24E−05
27	*Frac*	FBgn0035798	*—*	6.92E−05
28	*Fra*	FBgn0011592	*NEO1*	7.35E−05
29	*TfIIA-L*	FBgn0011289	*GTF2A1*	7.56E−05
30	*AdSL*	FBgn0038467	*ADSL*	7.56E−05
31	*CG15784*	FBgn0029766	*—*	7.89E−05
32	*CG7781*	FBgn0032021	*—*	7.90E−05
33	*CG15544*	FBgn0039804	*—*	8.17E−05
34	*Sfl*	FBgn0020251	*NDST2*	8.20E−05
35	*CG34354*	FBgn0085383	*TIA1/L1*	8.80E−05
36	*CG16812*	FBgn0032488	*C19orf47*	9.02E−05
37	*Px*	FBgn0003175	*—*	9.19E−05
38	*Pino*	FBgn0016926	*—*	9.29E−05
39	*Drat*	FBgn0033188	*—*	9.39E−05
40	*robo2*	FBgn0002543	*ROBO1/3*	9.66E−05

Modifiers are ranked based on the most significant SNP associated with each gene. Human orthologs include the ortholog with the greatest DIOPT score (≥5). Candidate modifiers above the double line reached a *P*-value threshold of *P* ≤ 1 × 10^−5^.

Due to the lack of significant variants that were identified with a standard *P*-value threshold of *P* ≤ 1 × 10^−5^, we also examined variants that fell within a more liberal threshold of *P* ≤ 1 × 10^−4^. Lowering the threshold does increase the false discovery rate (FDR) from 0.001 to 0.01%, but we still expect the variants in this less stringent pool to be enriched for true positives. With an arbitrary *P*-value cutoff of *P* ≤ 1 × 10^−4^, the GWA analysis identified 110 polymorphisms associated with stress preconditioning. Of these 110 polymorphisms, 81 fell within 1 kb of a known gene (Supplementary Table 1). These 81 variants included 7 downstream and 4 upstream of a gene, 3 in a 3′ UTR, 1 in a 5′ UTR, 56 in an intron, and 9 in protein-coding exons. Of the 9 in protein-coding exons, 1 is a nonsynonymous variant (*CG15784*), and 8 are synonymous. These 81 polymorphisms are associated with 40 unique *Drosophila* genes (Table 1). Of these 40 genes, 22 have a human orthologue with a DIOPT score of at least 5 (Hu *et al.* 2011). The 40 genes do not include any canonical ER stress or heat shock response genes, reinforcing the observation that differences in response to these individual stresses do not drive the variation in preconditioning. Gene ontology (GO) analysis did not identify any significant enrichment.

With our less stringent *P*-value cutoff, a subset of candidate modifiers identified in our GWA analysis indicated the potential involvement of chromatin organization and transcriptional regulation in preconditioning mechanisms. The GWA analysis identified *Tpl94D* (no human ortholog), an HMG-box domain protein with known roles in chromatin reorganization, indicating a potential role of chromatin organization in preconditioning outcomes (Gärtner *et al.* 2015). A second general transcription factor was also identified, *TfIIA-L* (*GTF2A1*). *TfIIA-L* (*GTF2A1*) is a member of the preinitiation complex required for RNA polymerase II initiation (Wang *et al.* 2020). The precise mechanism of how *Pdp1* (*HLF*) and *TfIIA-L* (*GTF2A1*) impact preconditioning is unclear, but their known roles suggest the possible importance of transcriptional regulation in preconditioning.

### GSEA uncovers the importance of histone methylation in preconditioning

Thus far, our investigation has only examined the candidate modifiers harboring individual polymorphisms that exceed a statistical threshold. Although this is a valuable method for identifying SNP-ranked candidate genes, it neglects most of the association data generated by our GWA analysis and relies on the discovery of highly significant individual SNPs. Using this method, very few candidate modifier genes were identified that were associated with the standard *P*-value cutoff of *P* ≤ 1 × 10^−5^. In order to obtain more biological insight from the full GWA output, we utilized GSEA to explore the entire association dataset (published on figshare).

Every polymorphism in the GWA dataset is assigned to a gene by the same method described above for GWA. GSEA then calculates a gene-based *P*-value enrichment score that determines the significance of all variants assigned to the gene (Subramanian *et al.* 2005; Palu *et al.* 2019; Talsness *et al.* 2020; Palu *et al.* 2022). The genes are then reranked by this new gene-based *P*-value. Each GO term is composed of a previously defined set of genes. Given this set of genes, GSEA asked if the genes in a given GO term are randomly distributed throughout the ranked list or found primarily at the top. GO terms with genes enriched at the top of the list are identified as enriched GO terms by GSEA.

**Fig. 2.**
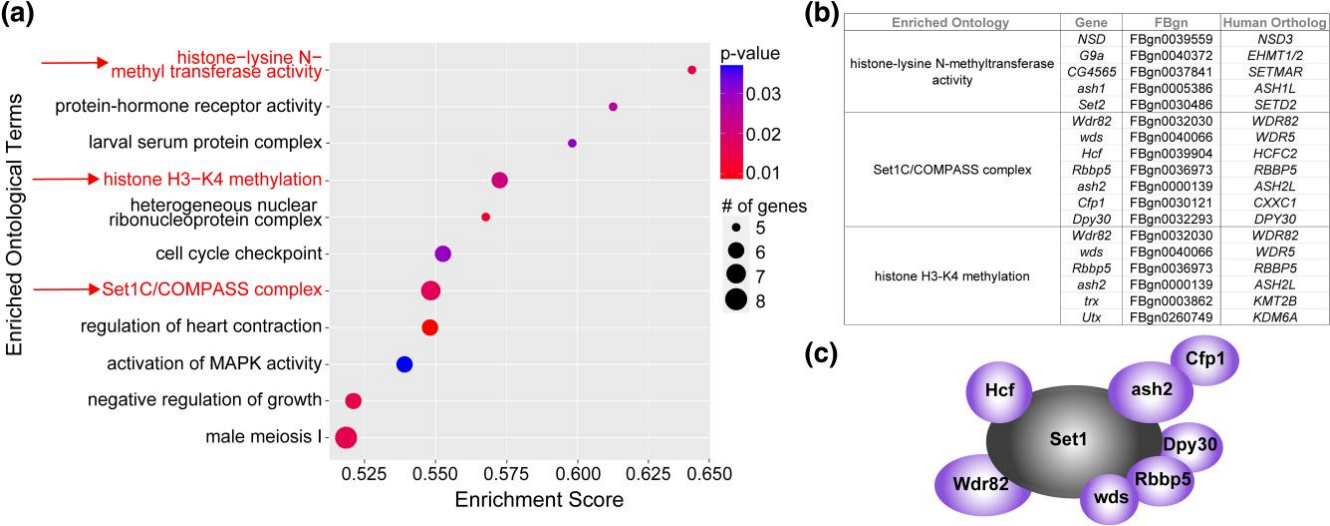
Gene set enrichment analysis (GSEA) reveals a role of histone methylation in preconditioning. a) Top GSEA results. Ontology terms are listed by descending enrichment score. *P*-values are illustrated by the red to blue color gradient. The number of genes contributing to each category is indicated by the size of the circle. Ontology terms related to histone methylation are shown in red and indicated by a red arrow. Cutoffs include *P*-value ≤ 0.05, number of genes ≥ 5, and enrichment score ≥ 0.50. b) Histone methylation ontology terms indicated in red are expanded to show the genes contributing to each ontology. FBgn and human ortholog (DIOPT score ≥ 5) are listed for each gene. c) The entire Set1/COMPASS complex is illustrated with all known subunits. All subunits with polymorphisms contributing to the ontology terms related to histone methylation are shown in purple.

There were 11 enriched ontology terms uncovered by GSEA (Fig. 2a; Supplementary Table 2) (*P* ≤ 0.05, enrichment score ≥ 0.50, number of genes contributing to ontology ≥ 5). Three gene ontology terms pointed to histone methylation as a key process regulating preconditioning (Fig. 2a, indicated terms highlighted). Each of the 3 histone methylation ontology terms contribute unique genes (Fig. 2b).

The most highly enriched ontological term is “histone-lysine N-methyltransferase activity” (GO:0018024). Of particular interest, the genes contributing to this enrichment include all the known *Drosophila* H3K36 methyltransferases, *NSD* (*NSD1-3*), *ash1* (*ASH1L*), and *Set2* (*SETD2*) (Wagner and Carpenter 2012; Bicocca *et al.* 2018; Huang and Zhu 2018) (Fig. 2b). These enzymes play roles in various processes, including transcription initiation and repression. Set2 (SETD2) associates with RNA polymerase II and plays a role in transcription elongation (Kizer *et al.* 2005).

Another enriched ontological term from our GSEA is “Set1C/COMPASS complex” (GO:0048188). Strikingly, the genes contributing to the enrichment of this category include all subunits of the *Drosophila* Set1 complex, except Set1 itself (Mohan *et al.* 2011) (Fig. 2b and c). Identifying nearly every subunit of the Set1 complex demonstrates that genetic variation in each component contributes to the observed variation in preconditioning outcomes, illustrating the importance of the Set1 complex in preconditioning. Set1 (SETD1A/B) is responsible for the majority of histone H3K4 trimethylation in *Drosophila* (Ardehali *et al.* 2011; Shilatifard 2012). H3K4me3 marks histones proximal to the promoters of actively transcribed genes and promotes efficient transcription initiation through interaction with RNA polymerase II (Ardehali *et al.* 2011; Shilatifard 2012).

The final enriched ontological term associated with methylation is “histone H3-K4 methylation” (GO:0051568). This ontological term includes many genes associated with Set1 (SETD1A/B) (Fig. 2b). Additionally, this ontology group includes *trx* (*KMT2A/B*), another enzyme associated with H3K4 histone methylation in *Drosophila* (Ardehali *et al.* 2011; Mohan *et al.* 2011; Shilatifard 2012). This category also includes *Utx* (*KDM6A*), an H3K27me3 demethylase linked to transcriptional regulation that colocalizes with RNA polymerase II (Smith *et al.* 2008; Van Der Meulen *et al.* 2014).

### RNAseq reveals potential gene expression predictors of preconditioning outcomes

Although GWA and GSEA uncovered a range of pathways that potentially underlie preconditioning mechanisms, the survival statistic is a culmination of many different processes. Expression differences in the basal state (no treatment), heat shock response, recovery postheat shock, and ER stress response might contribute to the ultimate survival outcome measured by the preconditioning screen. Thus, we sought to identify factors at early time points that might predict the outcome of preconditioning.

To identify genes with expression patterns predictive of preconditioning outcomes, we focused on the DGRP strains at the extreme ends of the distribution (Fig. 1b). These strains included 5 of the most beneficial preconditioning outcomes (RAL69, RAL93, RAL359, RAL387, RAL409) and 5 of the most detrimental preconditioning outcomes (RAL195, RAL304, RAL335, RAL737, RAL819). We refer to these groups as the beneficial and detrimental groups, respectively. To identify a standard mechanism for detrimental or beneficial preconditioning outcomes, we treated the 5 strains in each group as technical replicates in the RNAseq analysis instead of investigating each strain individually. Combining 5 genetically unique strains into a single group will wash out unique expression changes that each extreme strain carries. Only genes with substantial, similar effects across all 5 strains, will be detected as differentially expressed between the beneficial and detrimental groups.

RAL409 did not cluster with the other beneficial strains, as illustrated in PCA plots (Supplementary Fig. 3a–c). Therefore, we removed RAL409 as an outlier for the following analyses. The PCA plots also reveal more variability in the detrimental group than in the beneficial group (Supplementary Fig. 3c and d). This clustering pattern suggests that there is likely a common mechanism underlying a beneficial preconditioning outcome and that there may be more strain-specific mechanisms for a detrimental effect.

**Fig. 3.**
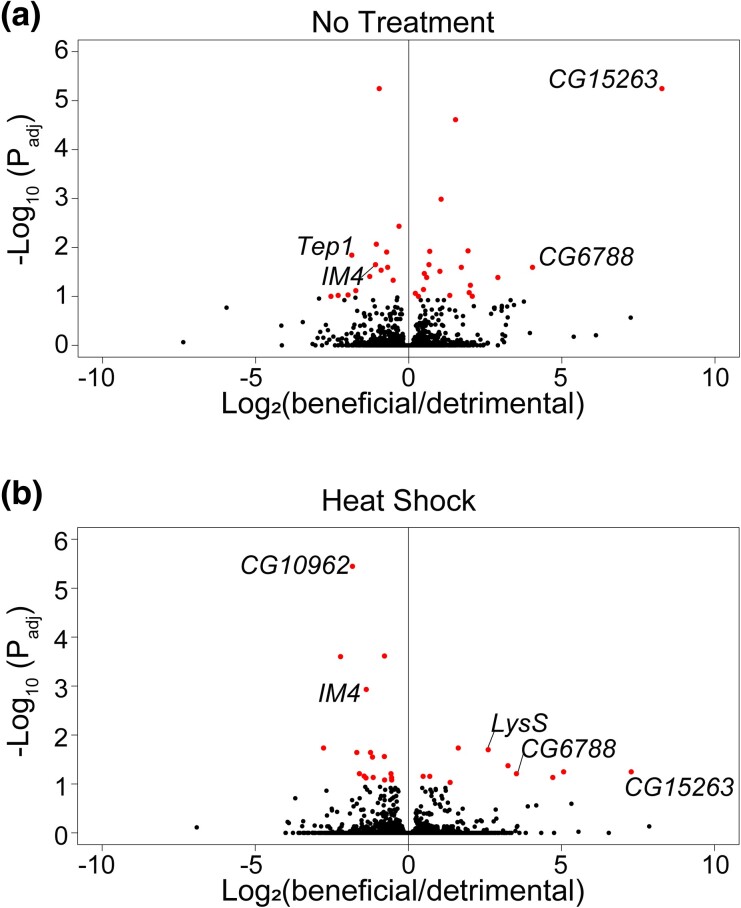
Differentially expressed genes in the beneficial group compared to the detrimental group. Volcano plots illustrating RNAseq results comparing beneficial/detrimental groups (a) with no stress or (b) immediately postheat shock. Each point represents a different gene. Red points are significantly differentially expressed using significance cutoffs of *P*_adj_ ≤ 0.10. Points to the right are upregulated in the beneficial group, and points to the left are downregulated. Labeled genes are discussed in the Results.

Under basal conditions, we found 19 genes upregulated and 14 genes downregulated in the beneficial group compared to the detrimental group (Fig. 3a, Table 2) (*P*_adj_ ≤ 0.10). The 2 most highly upregulated genes in the beneficial group are *CG15263* (no human ortholog) and *CG6788* (*FIBCD1*) (Log_2_(fold change) = 8.27 and 4.04, respectively) (Hu *et al.* 2011). While the function of *CG6788* is unknown, loss of function mutations in *FIBCD1* is associated with neurodevelopmental disorders in humans (Fell *et al.* 2022). *CG6788* may also play a role in neurodevelopment in *Drosophila.* Two of downregulated genes in the beneficial group, *Tep1 (CD109)* and *IM4* (no human ortholog) (Log_2_(fold change) = −1.86 and −1.09, respectively), participate in the *Drosophila* immune response by activating Toll (Dostálová *et al.* 2017; Cohen *et al.* 2020). These results suggest that basal immune status may affect whether preconditioning is beneficial or detrimental.

**Table 2.** Differentially expressed genes in the beneficial group with no treatment.

	Gene	FBgn	Human ortholog	Log_2_(fold change)	*P*-value	*P*_adj_
Upregulated in beneficial group	*CG15263*	FBgn0028853	*—*	8.27	7.91E−10	5.68E−06
	*CG6788*	FBgn0030880	*FIBCD1*	4.04	2.66E−05	2.55E−02
	*CR45860*	FBgn0267520	*—*	2.91	6.01E−05	4.11E−02
	*LysS*	FBgn0004430	*LYZ*	2.07	2.15E−04	9.94E−02
	*CG43829*	FBgn0261504	*—*	2.01	9.49E−05	5.93E−02
	*CR32864*	FBgn0000003	*—*	1.97	1.52E−04	8.38E−02
	*CR44066*	FBgn0264875	*—*	1.94	5.72E−06	1.17E−02
	*CG43829*	FBgn0264377	*—*	1.72	2.34E−05	2.55E−02
	*CG11912*	FBgn0031248	*—*	1.53	5.12E−09	2.45E−05
	*Jon99Fii*	FBgn0039777	*—*	1.34	2.00E−04	9.56E−02
	*CG3348*	FBgn0040609	*—*	1.06	2.88E−07	1.03E−03
	*CG16762*	FBgn0035343	*—*	1.02	3.64E−05	3.07E−02
	*CG18132*	FBgn0031345	*—*	0.69	6.69E−06	1.20E−02
	*Poc1*	FBgn0036354	*POC1A*	0.66	1.88E−05	2.25E−02
	*CG31343*	FBgn0051343	*TRHDE*	0.59	5.86E−05	4.11E−02
	*CG8560*	FBgn0035781	*CPA6*	0.51	4.27E−05	3.41E−02
	*CG17560*	FBgn0038450	*FAR2*	0.48	1.21E−04	7.23E−02
	*NAAT1*	FBgn0029762	*SLC6A5*	0.32	2.26E−04	9.98E−02
	*CG31523*	FBgn0051523	*ELOVL7*	0.21	1.63E−04	8.66E−02
Downregulated in beneficial group	*Or22a*	FBgn0026398	*—*	−2.54	2.29E−04	9.98E−02
	*CR44204*	FBgn0265099	*—*	−2.31	1.99E−04	9.56E−02
	*CR45959*	FBgn0267621	*—*	−1.98	1.82E−04	9.35E−02
	*Tep1*	FBgn0041183	*CD109*	−1.86	1.00E−05	1.44E−02
	*CR43304*	FBgn0262996	*—*	−1.73	1.33E−04	7.63E−02
	*CG10962*	FBgn0030073	*DHRS11*	−1.27	5.18E−05	3.91E−02
	*IM4*	FBgn0040653	*—*	−1.09	1.82E−05	2.25E−02
	*CG33282*	FBgn0053282	*SLC2A6*	−1.06	3.59E−06	8.59E−03
	*CG12917*	FBgn0033490	*EXOG*	−0.96	7.23E−10	5.68E−06
	*CR46007*	FBgn0267671	*—*	−0.90	3.25E−05	2.92E−02
	*CG14125*	FBgn0036232	*—*	−0.72	7.78E−06	1.24E−02
	*Nvy*	FBgn0005636	*CBFA2T3*	−0.69	2.54E−05	2.55E−02
	*CG15083*	FBgn0034399	*—*	−0.51	7.17E−05	4.68E−02
	*Px*	FBgn0003175	*—*	−0.32	1.28E−06	3.69E−03

Upregulated or downregulated genes in the beneficial group compared to the detrimental group. Genes are ranked by Log_2_(fold change). Genes with *P*_adj_ ≤ 0.10 are reported. Human orthologs have a minimum DIOPT score of 5.

Postheat shock, 10 genes are upregulated, and 17 genes are downregulated in the beneficial group compared to the detrimental group (Fig. 3b, Table 3) (*P*_adj_ ≤ 0.10). One of upregulated genes, *LysS (LYZ)* (Log_2_(fold change) = 2.61), and 1 downregulated gene, *IM4* (Log_2_(fold change) = −1.37), are implicated in immunity (Cohen *et al.* 2020; Marra *et al.* 2021; Qian *et al.* 2021). Of note, comparing between the 2 RNAseq analyses (no treatment and postheat shock), we found 7 differentially expressed genes that overlapped: *CG15263, CG6788* (*FIBCD1*), *LysS* (*LYZ*), and *Poc1* (*POC1A*) remain upregulated genes in the beneficial group, and *CR44204, CG10962* (*DHRS11*), and *IM4* remain downregulated, regardless of treatment (no stress or postheat stress). *CG15263* has no established function or human ortholog.

**Table 3.** Differentially expressed genes in beneficial group postheat shock.

	Gene	FBgn	Human ortholog	Log_2_(fold change)	*P*-value	*P*_adj_
Upregulated in beneficial group	*CG15263*	FBgn0028853	*—*	7.28	4.73E−05	5.66E−02
	*CR43488*	FBgn0263499		5.07	4.76E−05	5.66E−02
	*TwdlD*	FBgn0039444	*—*	4.72	9.43E−05	7.37E−02
	*CG6788*	FBgn0030880	*FIBCD1*	3.53	6.04E−05	6.17E−02
	*CR45994*	FBgn0267656		3.25	3.06E−05	4.24E−02
	*LysS*	FBgn0004430	*LYZ*	2.61	8.41E−06	2.00E−02
	*CG4815*	FBgn0039568	*—*	1.63	6.66E−06	1.84E−02
	*CG11131*	FBgn0037204	*—*	1.36	1.51E−04	9.31E−02
	*Poc1*	FBgn0036354	*POC1A*	0.70	8.14E−05	6.99E−02
	*CG12674*	FBgn0031388	*—*	0.48	8.41E−05	6.99E−02
	*CR44204*	FBgn0265099	*—*	−2.77	6.55E−06	1.84E−02
Downregulated in beneficial group	*Obp99b*	FBgn0039685	*—*	−2.21	4.52E−08	2.50E−04
	*CG10962*	FBgn0030073	*DHRS11*	−1.82	2.15E−10	3.58E−06
	*BomBc3*	FBgn0040582	*—*	−1.68	1.18E−05	2.28E−02
	*DptB*	FBgn0034407	*—*	−1.60	5.60E−05	6.17E−02
	*CR44552*	FBgn0265745	*—*	−1.44	7.49E−05	6.92E−02
	*Gnmt*	FBgn0038074	*GNMT*	−−1.38	1.09E−04	7.54E−02
	*IM4*	FBgn0040653	*—*	−1.37	2.82E−07	1.17E−03
	*Srg1*	FBgn0039239	*—*	−1.23	1.23E−05	2.28E−02
	*CG33509*	FBgn0053509	*—*	−1.17	1.87E−05	2.83E−02
	*Dso2*	FBgn0067905	*—*	−1.15	9.75E−05	7.37E−02
	*Fon*	FBgn0032773	*—*	−0.78	1.66E−05	2.75E−02
	*CR44754*	FBgn0265967	*—*	−0.78	2.93E−08	2.43E−04
	*CG7381*	FBgn0038098	*—*	−−0.78	1.29E−04	8.30E−02
	*Mes2*	FBgn0037207	*—*	−0.57	6.31E−05	6.17E−02
	*Uif*	FBgn0031879	*—*	−0.55	1.03E−04	7.44E−02
	*Elal*	FBgn0013949	*—*	−0.54	1.30E−04	8.30E−02

Upregulated or downregulated genes in the beneficial group compared to the detrimental group. Genes are ranked by Log_2_(fold change) within their group. Genes with *P*_adj_ ≤ 0.10 are reported. Human orthologs have a minimum DIOPT score of 5.

Another downregulated gene postheat shock was *CG10962* (*DHRS11*) (Log_2_(fold change) = −1.82), which may play a role in the ER stress response. A previous DGRP GWA study found that a SNP in *CG10962* (*DHRS11*) is significantly associated with changes in survival time in an environment of constant ER stress (Chow *et al.* 2013). *CG10962* (*DHRS11*) is the only potential predictor of preconditioning outcomes associated with ER stress. We did not identify any significant ER stress or heat shock genes in this analysis, which reinforced the hypothesis that the individual stresses of preconditioning do not play a role in preconditioning mechanisms.

### Loss of *Set1* leads to increased variance in preconditioning outcomes

**Fig. 4.**
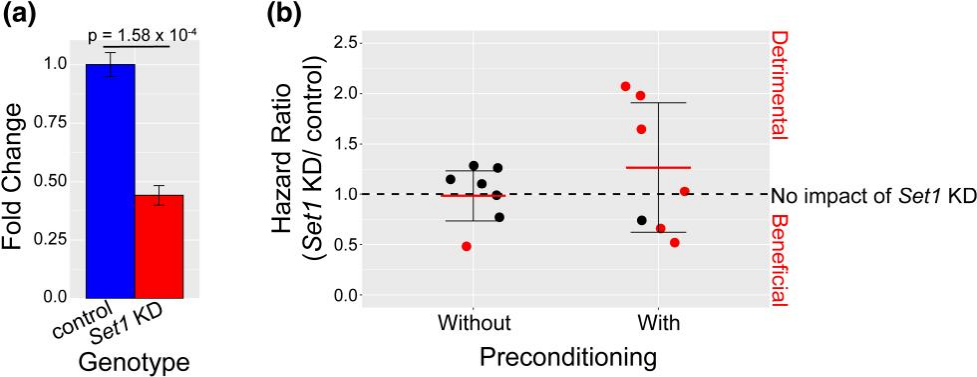
Impact of *Set1* KD on stress preconditioning. a) *Set1* RNAi results in ∼55% reduction in *Set1* expression. b) Plot of the hazard ratios comparing *Set1* KD to its genetically matched control without and with preconditioning. Each point represents the hazard ratio from a different replicate of 100 *Set1* KD and control flies. A hazard ratio < 1 indicates *Set1* KD had a beneficial effect compared to the control, a hazard ratio = 1 indicates no effect, and a hazard ratio > 1 indicates a detrimental effect. The dashed line indicates no effect (hazard ratio = 1) of *Set1* KD. The red line indicates the mean, and the cross bars indicate ±1 standard deviation. Red points indicate a statistically significant hazard ratio.

Our preconditioning screen and GWA analysis uncovered histone methylation and transcriptional regulation as candidate pathways for modifying responses to preconditioning (Table 1; Fig. 2). We focused our initial functional investigation on *Set1* (*SETD1A/B*) because all subunits bound to Set1 contain genetic variants contributing to preconditioning outcomes (Fig. 2c). *Set1* is highly conserved from yeast to humans, is responsible for the majority of H3K4 trimethylation in *Drosophila*, and is critical for the optimal transcription of active genes (Ardehali *et al.* 2011; Brown David *et al.* 2017). Loss of *Set1* or its subunits leads to widespread impacts on gene expression (Brown David *et al.* 2017). We decided to focus on *Set1* because it is the central component of this complex and because ubiquitous RNAi knockdown of SET1 complex subunits resulted in lethality. We used ubiquitous expression of *Set1* RNAi to knockdown *Set1* (tubulin-GAL4 driver, “*Set1* KD”). This reduced *Set1* expression by approximately 55% (Fig. 4a, Supplementary Table 3) and resulted in no lethality. The DGRP is a collection of inbred lines derived from a natural population. Therefore, any polymorphisms found using the DGRP must be tolerated, nonlethal, and not eliminated during the inbreeding process (Mackay *et al.* 2012). This milder reduction in *Set1*, but not complete loss, better models the small effect sizes of natural variants identified in the preconditioning screen.

To investigate the impact of *Set1* KD on preconditioning, we utilized the same experimental design as the stress preconditioning screen (Fig. 1a). In place of DGRP strains, we performed the assay using *Set1* KD and a genetically matched control. We utilized the Cox proportional hazards model to generate a hazard ratio that compares *Set1* KD to the control with and without preconditioning (Fig. 4b, Supplementary File 2). We first tested whether *Set1* KD affects survival under ER stress without preconditioning. A hazard ratio of 1 indicates no significant change in ER stress survival without preconditioning between *Set1* KD and the control. Of 7 replicates, only 1 replicate showed an effect (hazard ratio = 0.57, *P*-value ≤ 0.05) (Fig. 4b). Therefore, *Set1* KD generally (6/7 replicates) does not impact ER stress survival times without preconditioning.

Next, we tested whether *Set1* KD affects survival under ER stress with preconditioning. More replicates show a significant effect due to the loss of *Set1* with preconditioning than without preconditioning. Four replicates result in a significant detrimental effect (hazard ratios = 2.14, 1.84, 1.76, 1.10, *P*-value ≤ 0.05), 1 results in no significant effect, and 2 replicates result in a significant beneficial effect (hazard ratios = 0.57, 0.57, *P*-value ≤ 0.05) of *Set1* KD on preconditioning outcomes. There was more variance in the impacts of *Set1* KD with preconditioning than without preconditioning (F test *P*-value = 0.037). These results indicate that *Set1* KD has a variable effect on preconditioning. However, it is possible that the phenotype we examined was not sufficiently precise to uncover the mechanism underlying this effect. Survival on TM is a cumulative measure that can be impacted by any step in the preconditioning assay. Therefore, if *Set1* KD has a subtle effect on any individual step of the preconditioning assay or opposing effects during different steps it may not translate to a measurable, consistent impact on the cumulative phenotype. We decided to investigate the impact of *Set1* KD at each individual step of the preconditioning assay in an effort to elucidate Set1's role in preconditioning.

### Loss of *Set1* leads to dysregulation of critical stress response genes during preconditioned ER stress

ER stress and heat stress require robust transcriptional responses to refold misfolded proteins and return to homeostasis (Guertin *et al.* 2010; Chow *et al.* 2013). *Set1* plays an active role in the efficient upregulation of stress response genes (Ardehali *et al.* 2011; Brown David *et al.* 2017). The dysregulation of these transcriptional responses could lead to adverse effects on preconditioning. Therefore, we hypothesized that *Set1* KD alters the transcription levels of critical stress response genes, leading to abnormal mRNA levels at 1 or more steps of the preconditioning assay.

The stress preconditioning assay utilizes survival as a phenotypic readout. This provides insight into the cumulative effect of *Set1* KD on preconditioned ER stress but does not reveal the individual contributions of each step of the preconditioning process (Fig. 1a). Thus, we investigated the role of *Set1* in preconditioning at each step of the stress preconditioning assay, including without stress (no treatment), immediately following a 30-min heat shock treatment (heat shock), immediately following a 4-h recovery from heat shock (postrecovery), and immediately following a 16-h TM treatment without and with preconditioning (ER stress and preconditioned ER stress, respectively).

**Fig. 5.**
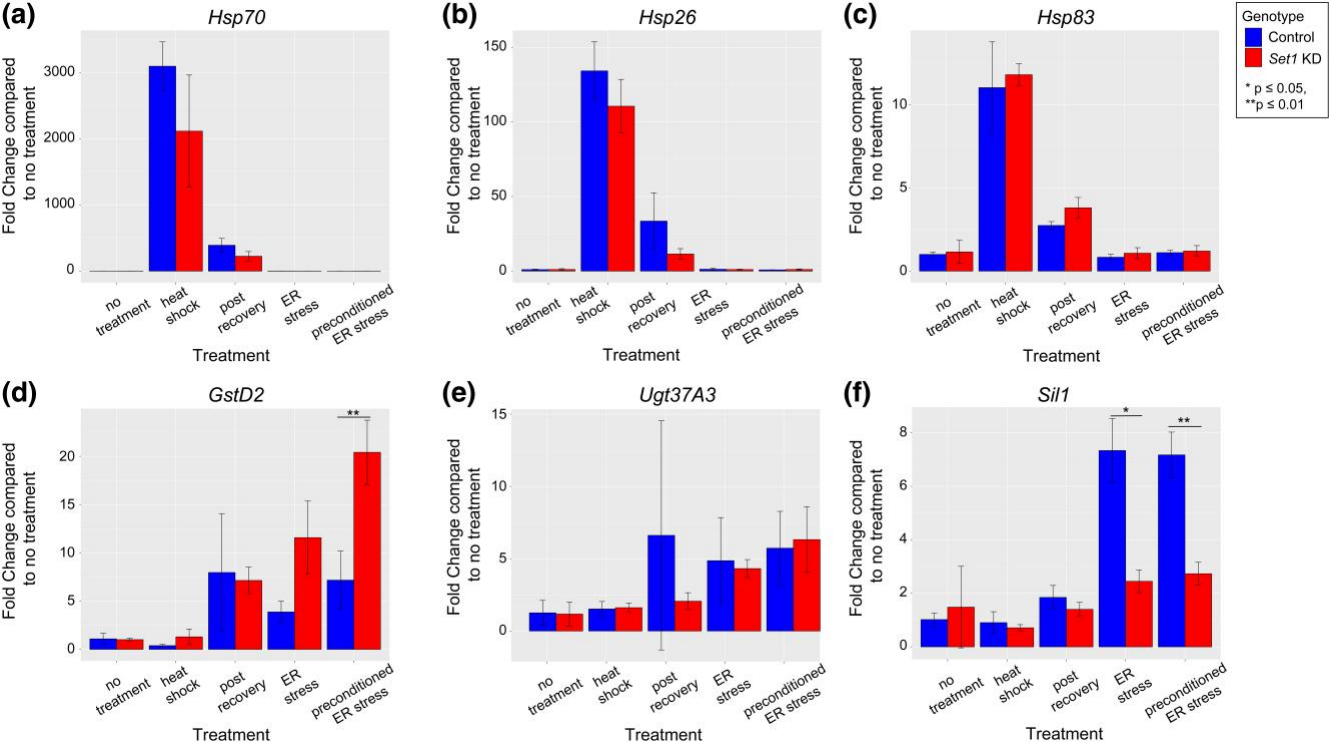
Set1 is necessary to regulate transcript levels of a subset of genes post-stress. Transcript expression change between controls (blue) and *Set1* KD (red). Paired t-test between control and *Set1* KD at each time point, significant differences are noted. a–c) Heat stress genes. d–f) ER stress genes.

We evaluated the role of *Set1* in upregulating canonical stress response genes at each stage of our preconditioning assay. We examined 3 established heat shock genes (*Hsp70, Hsp26, Hsp83*) (Guertin *et al.* 2010; Ardehali *et al.* 2011) and 3 established ER stress genes (*Sil1, Ugt37A3, GstD2)* (Chow *et al.* 2013). In our investigation of Set1's role in preconditioning mechanisms, we focused on selecting heat shock and ER stress genes that were likely to interact with Set1. Our choice of *Hsp70*, *Hsp26*, and *Hsp83* was based on previous studies that demonstrated connections between Set1 and the transcription of these 3 heat shock genes (Guertin *et al.* 2010; Ardehali *et al.* 2011). Considering that H3-K4 histone methylation is found near the promoters of actively transcribed genes, our goal was to assess genes with high expression levels during ER stress (Ardehali *et al.* 2011; Chow *et al.* 2013). Given Set1's impact on actively transcribed genes (Ardehali *et al.* 2011), we specifically selected *Sil1*, *Ugt37A3*, and *GstD2*. These choices were informed by their status as the 3 most highly expressed genes following Chow *et al*. (2003)'s 8-h feeding of TM, a method similar to ours, based on the average expression calculated across all 20 DGRP strains. We hypothesized that loss of *Set1* would lead to disrupted transcript levels of these critical stress response genes after 1 or more steps of the preconditioning assay, ultimately disrupting preconditioning outcomes. We first examined the heat shock and ER stress genes’ transcriptional responses to stress in control flies (Supplementary Fig. 4). Next, we examined the transcriptional response of the same 6 genes in *Set1* KD flies exposed to the same preconditioning stress paradigm (Supplementary Fig. 4). After examining how stress impacts these genes within each genotype, we analyzed the effect of genotype on transcriptional responses at each time point (control vs *Set1* KD) (Fig. 5). This allowed us to measure how *Set1* KD altered gene expression throughout the preconditioning assay compared to the control.

None of the heat shock genes examined (*Hsp70, Hsp26, Hsp83*) were significantly impacted by *Set1* KD compared to the control at any point of the preconditioning assay (Fig. 5a–c). They all showed a robust response to heat shock. This suggests that *Set1* is not necessary for normal heat shock gene expression during preconditioning. Similar to the heat shock genes, the ER stress gene *Ugt37A3* showed no significant impact of *Set1* knockdown across the 5 time points examined (Fig. 5e).

In contrast, during preconditioned ER stress, *Set1* KD resulted in the misregulation of the ER stress genes *GstD2* and *Sil1*. *GstD2* and *Sil1* expression were not significantly altered by loss of *Set1* at any earlier timepoints. Following preconditioned ER stress, *GstD2* was upregulated in *Set1* KD flies more than in controls (Fig. 5d; *Set1* KD: 20.42 ± 3.33; control: 7.17 ± 3.01-fold; *P* ≤ 0.01). *Sil1* displayed significantly lower expression in *Set1* KD flies compared to controls after ER stress without preconditioning (Fig. 5f; *Set1* KD: 2.45 ± 0.43; control: 7.34 ± 1.19; *P* ≤ 0.05) and with preconditioning (Fig. 5f; *Set1* KD: 2.73 ± 0.43; control: 7.18 ± 0.85; *P* ≤ 0.01). Taken together, our results suggest that *Set1* is necessary to regulate normal expression of a subset of critical ER stress response genes during preconditioning. To further investigate and validate this result, future studies would benefit from more extensive assays that evaluate *Set1* knockdown's impact across the entire transcriptome or chromatin landscape during preconditioning.

## Discussion

Organisms require an effective ER stress response for healthy development and aging. Disruption of the ER stress response underlies many human diseases—from diabetes to neurodegeneration (Chaudhuri and Paul 2006; Back and Kaufman 2012; Brown and Naidoo 2012; Cnop *et al.* 2012; Labbadia and Morimoto 2015). Therefore, a comprehensive understanding of the ER stress response's fundamental biology is critical for informing future therapeutic development. The ER stress response naturally occurs within a complex history of other stresses that impact the response. Preconditioning is the phenomenon of transient exposure to stresses affecting responses to subsequent stresses. Research investigating the ER stress response has primarily focused on isolated stress events. To investigate how preconditioning impacts the canonical ER stress response, we utilized natural genetic variation in *Drosophila* to perform an unbiased screen.

We found that preconditioning outcomes vary greatly, depending on genetic background. Preconditioning outcomes in the literature primarily report a beneficial impact of preconditioning, but we found that this outcome is genotype-specific. Although several DGRP strains displayed a beneficial preconditioning outcome, many show neutral or detrimental effects. The spectrum of unique preconditioning outcomes in our screen made it possible to identify several candidate pathways that drive variation in outcomes. We hypothesize that previous studies reported mainly a beneficial effect because of the use of a single laboratory strain and the positive report bias in scientific literature. In many cases, these strains are lab adapted for hundreds of generations and that may affect the preconditioning response. Discovering unexpected biological connections is a significant advantage of performing an unbiased genetic screen. First, our association analysis generated 40 candidate modifier genes of preconditioning. The list of candidate modifiers included 2 general transcriptional factors, *Pdp1 (HLF)* and *TfIIA-L (GTF2A1)*, and 1 gene involved in chromatin organization, *Tpl94D* (no human ortholog). Second, our GSEA identified multiple pathways that harbor genetic variants that impact preconditioning outcomes. The GSEA results included several ontologies involved in histone methylation. These results suggest a connection between transcriptional regulation, histone methylation, and preconditioning. Third, our RNAseq experiment uncovered 53 genes whose gene expression patterns may predict preconditioning outcomes.

These 3 different approaches did not identify any canonical ER stress or heat shock genes, which illustrates that the pathways underlying preconditioning are separate from the canonical pathways of the individual stressors. We chose to use 2 unique stressors in our preconditioning model in an effort to prioritize studying the effect of preconditioning over the effects of any single stressor. The use of 2 stressors is a commonly used approach to study preconditioning (Hayashi *et al.* 2003; Hung *et al.* 2003; Kennedy *et al.* 2014; D’Urso *et al.* 2016; Labbadia *et al.* 2017). If our results included several canonical ER stress or heat shock genes than further experimentation would be required to tease apart whether the gene was impacting preconditioning as a whole or the organism's ability to respond to 1 of the individual stressors. Since we did not identify these genes through our preconditioning model and DGRP screen results did not correlate with experiments studying either singular stress (Supplementary Fig. 2), we believe our preconditioning model is specifically examining preconditioning effects.

Our GWAS, GSEA, and RNAseq results had less overlap than we anticipated. We had hypothesized that a subset of the genes and pathways identified in our GWAS and GSEA would also be detected as differential expressed genes in the early stages of the preconditioning model. Our results did not point towards a single strong candidate gene identified in all 3 analyses. This may be because whatever differentiates the DGRP strains with beneficial vs detrimental preconditioning outcomes is not reflected at the transcript level. It may be that the genes and pathways involved in the overall preconditioning effects detected by our screen are distinct from genes involved in the earlier timepoints isolated for RNAseq. This lack of overlap may not be entirely surprising, as expression changes can be quite downstream from a variant identified by GWAS. For example, in previous studies of ER stress, we found little overlap between RNAseq results and GWAS hits (Chow *et al.* 2013 (PNAS)). It is also possible that we would better detect a relationship between expression and genetic variants if we incorporated an eQTL analyses in the future. Although there was less overlap than we predicted between experiments, each method provided unique insights into the underlying mechanism of preconditioning.

Our unbiased approach revealed many intriguing new avenues for exploration. The association analyses identified 2 heparan sulfate sulfotransferases, *Hs6st* (*HS6ST1*) and *sfl* (*NDST2*). Heparan sulfate genes play a role in the cellular response to misfolded proteins (He *et al.* 2014). Candidate modifier genes also included 2 genes involved in lipid homeostasis, *LpR2* (*VLDLR*) and *CG17097* (*LIPA/F/K/M/N*)*. LpR2* (*VLDLR*) is involved in neutral lipid uptake into cells, and *CG17097* (*LIPA/F/K/M/N*) is a triacylglycerol lipase involved in lipid metabolism and uptake (Cermelli *et al.* 2006; Parra-Peralbo and Culi 2011). We previously demonstrated that disrupting cellular fatty acid composition through the disruption of *Baldspot/ELOVL6* impacts the ER stress response in *Drosophila* (Palu and Chow 2018). The differentially expressed genes between beneficial and detrimental preconditioning groups included a previously unknown connection between preconditioning and immune pathways. Interactions between immune and stress responses have been previously observed (Davies *et al.* 2012). For example, immune peptides have been reported to block apoptosis signals triggered by stress response pathways (Krautz *et al.* 2020).

Preconditioning has an established role in providing robust neuronal protection from subsequent brain injuries after initial stress (Hayashi *et al.* 2003; Stetler *et al.* 2014). In one instance, a paradigm of heat stress protected rat brains from neuronal death by localized ischemia (Xu *et al.* 2002). Although many cases of preconditioning lead to neuroprotection, a genetic link between neurological function and preconditioning is unknown. Our association analysis identified several candidate modifier genes of preconditioning that encode proteins with known roles in axon guidance and neural development, including *loaf* (no human ortholog) (Douthit *et al.* 2021), *ckn* (no human ortholog) (Weng *et al.* 2011), *sidestep* (no human ortholog) (Siebert *et al.* 2009), *frac* (no human ortholog) (Miller *et al.* 2011), *fra* (*NEO1*) (Akin and Zipursky 2016), and *robo2* (*ROBO1/3*) (Simpson *et al.* 2000). Future studies are required to elucidate the underlying mechanisms of preconditioning in the brain.

We prioritized *Set1 (SETD1A/B*) for our initial functional validation because the association analysis and GSEA both indicated a potential role of chromatin organization and transcriptional regulation in preconditioning. Although the analyses identified several histone methylation genes, the Set1/COMPASS complex was the only candidate with genetic variants in all its subunits, except Set1 itself. The Set1/COMPASS complex marks promoter-proximal histones at actively transcribed genes. Set1 is a histone H3 lysine 4 (H3K4) methylase highly conserved from yeast to humans (Ardehali *et al.* 2011; Shilatifard 2012). Yeast, *Drosophila*, and humans have 1, 3, and 6 Set1/COMPASS family members, respectively (Ardehali *et al.* 2011; Shilatifard 2012). In *Drosophila, Set1* is responsible for the bulk of H3K4 di- and trimethylation but the other 2 Set1/COMPASS family members, Trr and Trx, contribute to di- and tri-methylation to a lesser extent (Ardehali *et al.* 2011).

The “stress memory” model of preconditioning proposes that cells acquire and retain epigenetic marks during initial stress, allowing them to “remember” the stress and respond more efficiently to subsequent stress (D’Urso *et al.* 2016; Lämke and Bäurle 2017; Fabrizio *et al.* 2019). This model provides a potential explanation of how Set1 is involved in preconditioning. In plants exposed to sequential droughts, transcript levels of a subset of stress-responsive genes are elevated more quickly during secondary dehydration, compared naive dehydration (Ding *et al.* 2012). After the drought state was resolved, these plants retained high levels of H3K4 trimethylation at the “primed” stress-responsive genes, and RNA polymerase II also stalled at these genes (Ding *et al.* 2012). Yeast previously exposed to salt stress has 77 genes that activate more rapidly when exposed to subsequent oxidative stress (D’Urso *et al.* 2016). During stress in yeast, actively transcribed genes acquire H3K4 di- and trimethylation marks, and genes that acquire a “memory” for stress retain H3K4 dimethylation after stress is resolved. Once again, these marks are associated with RNA polymerase II binding and retention at promoters. In vertebrates, the loss of Set1/COMPASS complex members leads to global misregulation of gene expression (Brown David *et al.* 2017). In human HeLa cells, hundreds of genes show a more rapid transcriptional upregulation in response to IFN-γ after previous exposure to IFN-γ, and this “memory” is associated with H3K4 dimethylation and poised RNA polymerase II at the promoters of these genes (Light *et al.* 2013). Set1 is required for transcriptional memory in yeast and human cells.

In *Drosophila*, Set1 is actively recruited to stress response genes following stress application (Ardehali *et al.* 2011). Loss of *Set1* leads to less efficient upregulation of *Hsp70* and *hsp83*. Our study did not recapitulate these results, most likely due to differences in our experimental design. Kusch *et al*. utilized *Drosophila* S2 cell lines and larval salivary glands instead of whole adult flies and assayed more time points postheat stress. Kusch *et al*. reported H3K4 trimethylation marks promoter-proximal to *Hsp70* and *hsp83* were significantly reduced in *Set1* KD cells following heat shock, and RNA polymerase II displayed increased stalling at these promotors. Disruption of RNA polymerase II kinetics, such as in promoter-proximal pausing, is known to lead to transcriptional dysregulation (Jonkers *et al.* 2014). The role of H3K4 dimethylation post-stress was not investigated in this study.

We established that disruption of *Set1* leads to dysregulation of a subset of stress response genes, particularly the ER stress genes. We propose that Set1 plays a role in preconditioning by establishing transcriptional “memory” of stress events. We hypothesize that in *Drosophila*, Set1 adds H3K4 methylation marks (di- and tri-) promoter-proximal to stress-responsive genes during stress, and a subset of these marks are retained and influence how RNA polymerase II interacts with these genes to alter future transcriptional responses. To validate this model, further investigations into the global transcriptional and epigenetic impacts of *Set1* KD during preconditioning will need to be explored.

## Supplementary Material

jkad265_Supplementary_Data

## Data Availability

Additional data files were uploaded to figshare and gene expression omnibus (GEO). The full stress preconditioning GWA results are located on figshare at: https://figshare.com/articles/dataset/StressPreconditioningDGRP_GWAoutput_txt/22266238. Fatsq files supporting the RNAseq experiments can be found at: https://www.ncbi.nlm.nih.gov/geo/query/acc.cgi?acc=GSE226958. GSEA code can be found on figshare at: https://doi.org/10.25387/g3.9808379. Additional code and protocols detailing how the code was used can be found at: https://github.com/kgowings/ER_stress_preconditioning/tree/main. Supplemental material available at G3 online.
