# Supplementary material for: A Drosophila screen identifies a role for histone methylation in ER stress preconditioning
Source: G3 (Bethesda). 2023 Dec 14;14(2):jkad265. doi: 10.1093/g3journal/jkad265 (PMC11021027; doi:10.1093/g3journal/jkad265)
Supplement: jkad265_Supplementary_Data [file jkad265_supplementary_data.zip › Supplemental_Material_Legends_G3-2023-404642.docx]

Supplemental Information

**Figure S1: P-values for variant association fall within the expected distribution for the linear mixed model.** Quantile-quantile plot was generated for P-values (-log_10_[P-value]) across all polymorphisms (MAF ≥ 0.05) tested in the stress preconditioning GWA. Calculated expected values are distributed along the x-axis, with observed values along with y-axis.

**File S1: Stress preconditioning screen results.** ER stress survival with and without preconditioning and hazard ratios are provided for each DGRP line, and tabs are grouped by strain**.** Each fly’s survival time on TM, treatment (preconditioning or control), and replicate group is provided. The first tab includes a summary of each strain's hazard ratio, p-value, and ln(hazard ratio).

**Figure S2: Correlation plots comparing stress preconditioning screen results to previously reported DGRP impacts on ER stress, heat tolerance, and longevity.** The distribution of stress preconditioning screen hazard ratios across DGRP strains is not correlated with (A) the distribution of the hazard ratio of death rates of 114 DGRP lines on TM-induced ER stress compared with drug-free control food(Chow et al. 2013); (B) the distribution of maximum heat tolerance of 100 DGRP lines(Lecheta et al. 2020); (C) the distribution of the mean lifespan of males across 186 DGRP lines at 25°C(Huang et al. 2020).

**Table S1: Top SNPs from GWA.** The top 81 SNPs from the GWA analysis are listed with chromosome location, FBgn ID, associated gene, variant type, distance from gene, major and minor allele, allele frequency, and significance. Cutoffs include: SNPs +/- 1 kb of a known gene, af ≥ 0.05, p ≤ 0.0001.

**Table S2: Top enriched GSEA categories.** GSEA categories are ranked in descending order by enrichment score. Genes with polymorphisms contributing to each ontology are indicated as FBgns. Cutoffs include p-value ≤ 0.05, number of genes > 4, and enrichment score ≥ 0.50.

**Figure S3: PCA plots of RNAseq results.** The color of each point indicates the treatment group, and the shape indicates the preconditioning group. Under each PCA plot, is a table detailing outlier calculation data. (A) PCA plot of RNAseq of five DGRP strains with beneficial preconditioning outcomes and five with detrimental outcomes without treatment. (B) PCA plot of RNAseq on the same strains as (A) immediately post-heat shock. (C) Heat shock versus no heat shock PCA plot for beneficial strains. (D) Heat shock versus no heat shock PCA plot for detrimental strains.

**Table S3: qPCR results.** Reports dCt and 2^-ddCT values for the following target genes: *Hsp70*, *Hsp26*, *Hsp83, Sil1*, *Ugt37A3*, *GstD2*, and *Set1*. The genotypes queried include *Set1* KD and Attp40 control. Flies were collected without treatment, immediately after heat shock, after a four-hour recovery, and after a 16-hour TM treatment with and without preconditioning. *Set1* was only investigated at the no treatment timepoint to evaluate knockdown efficiency.

**File S2: Set1 KD stress preconditioning assay lifespan data.** The first tab includes a summary of each replicate's hazard ratios (Set1 KD/ control with and without preconditioning), and p-value. The following tabs include the survival time on TM on hours, genotype, heat shock treatment, and vial for each fly in each replicate. Each tab is a different replicate.

**Figure S4: Analysis of stress response gene expression post-stress relative to no treatment.** Each plot illustrates qPCR fold change data for one of six stress response genes in control (blue) or *Set1* KD (red) flies. (A-C) Genes upregulated immediately after heat stress. (D-F) Genes upregulated post-ER stress.
