# Supplementary figures and images for: A Drosophila screen identifies a role for histone methylation in ER stress preconditioning
Source: G3 (Bethesda). 2023 Dec 14;14(2):jkad265. doi: 10.1093/g3journal/jkad265 (PMC11021027; doi:10.1093/g3journal/jkad265)

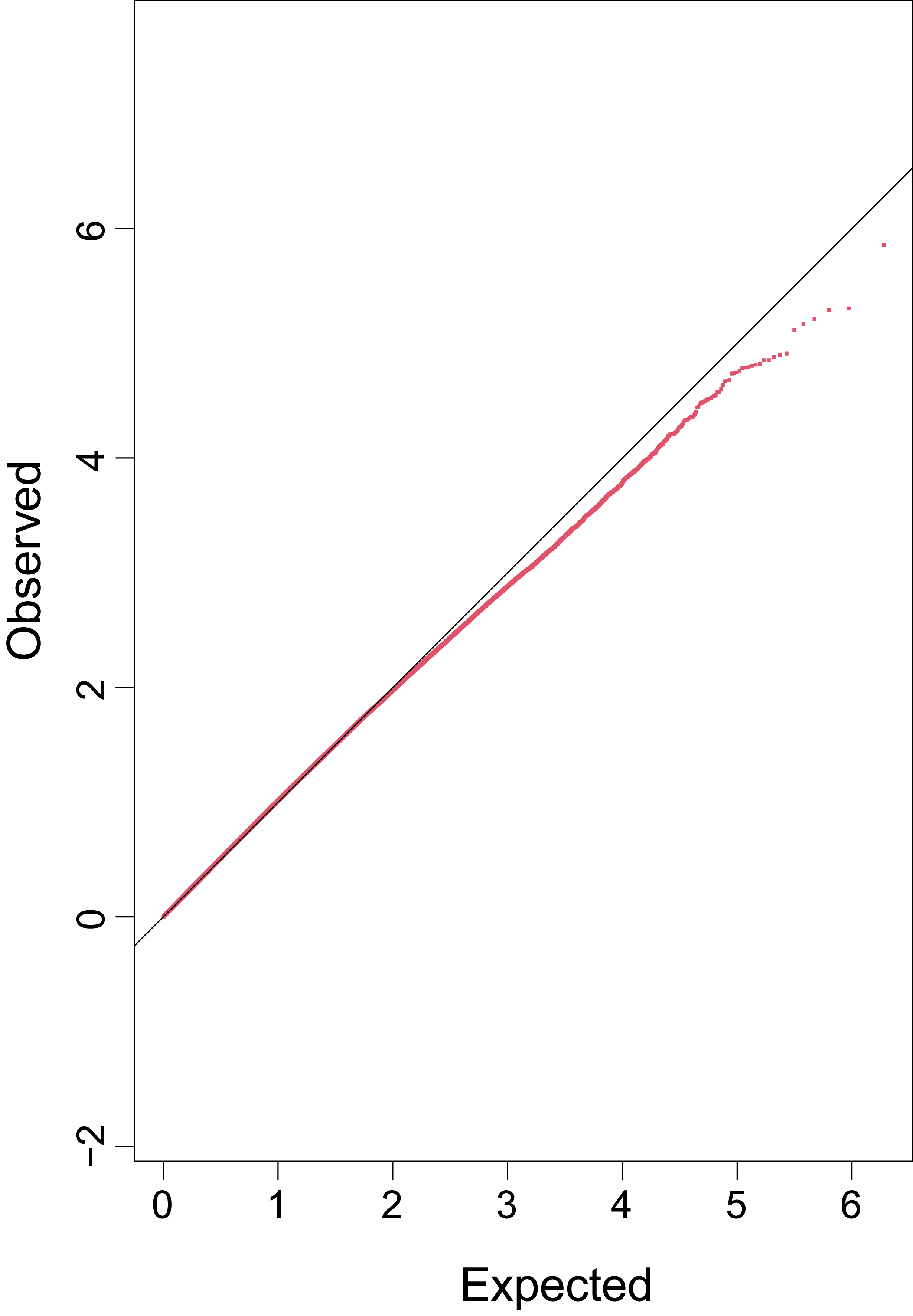

Supplement: jkad265_Supplementary_Data [file jkad265_supplementary_data.zip › Figure_S1_G3-2023-404642.tif]

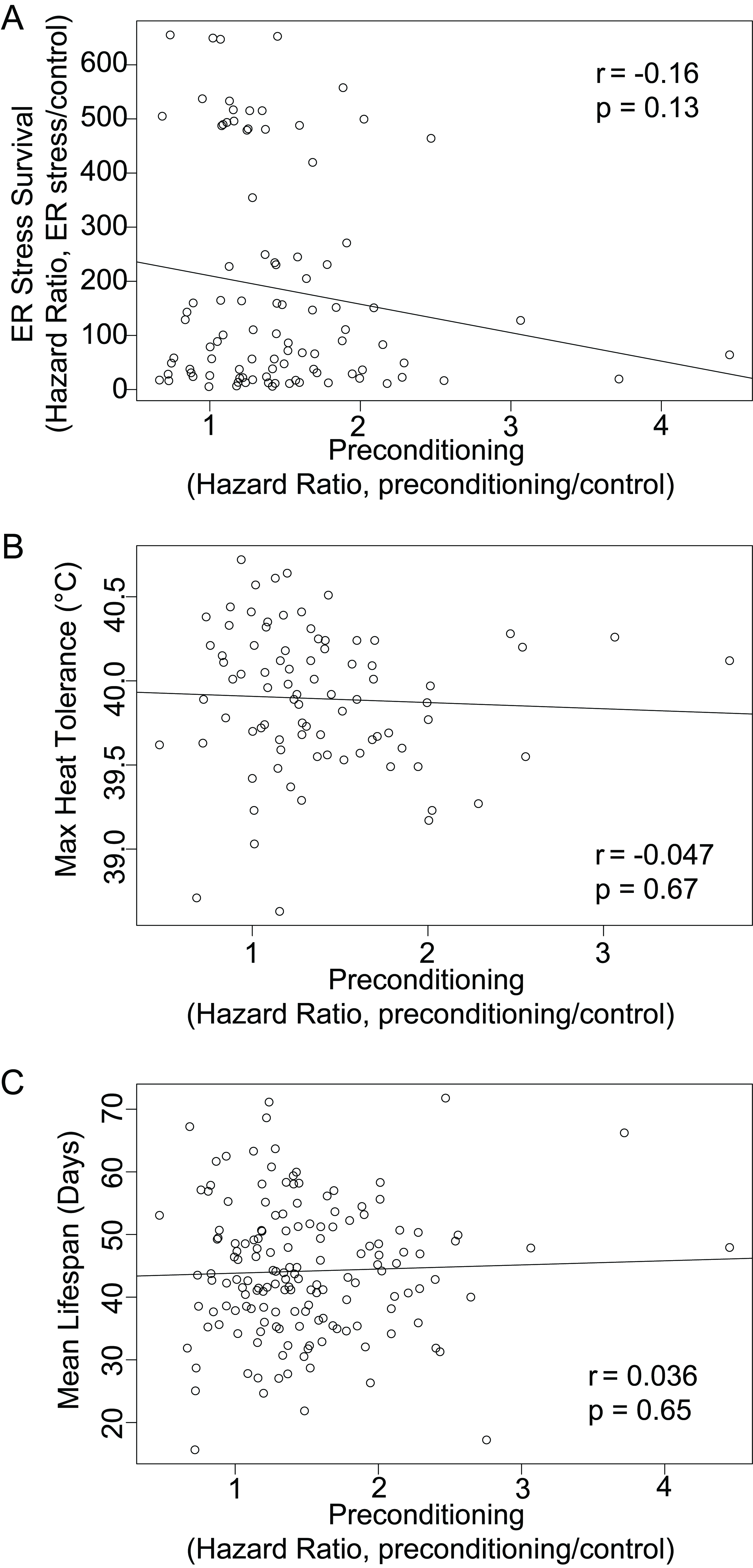

Supplement: jkad265_Supplementary_Data [file jkad265_supplementary_data.zip › Figure_S2_G3-2023-404642.tif]

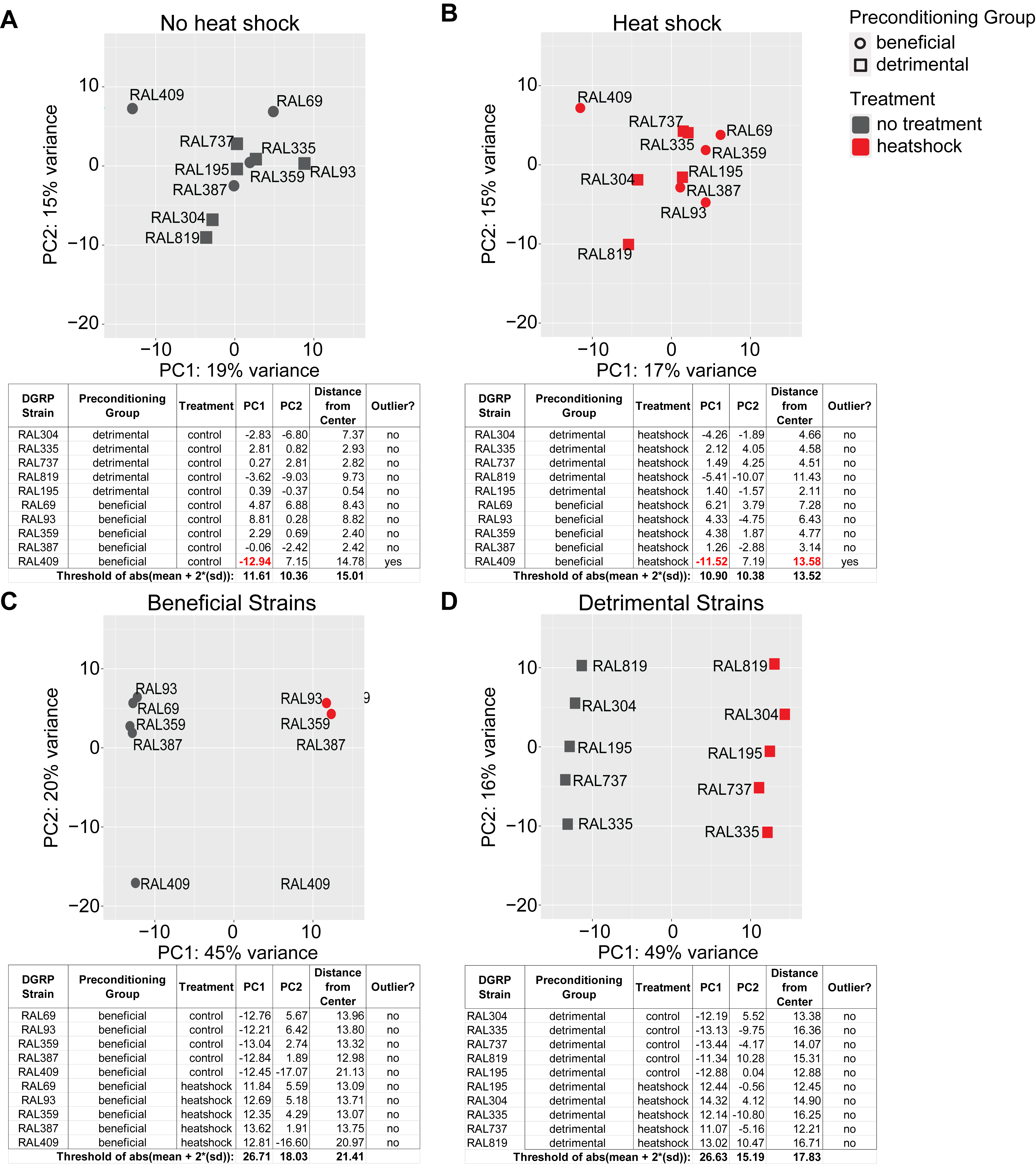

Supplement: jkad265_Supplementary_Data [file jkad265_supplementary_data.zip › Figure_S3_G3-2023-404642.tif]

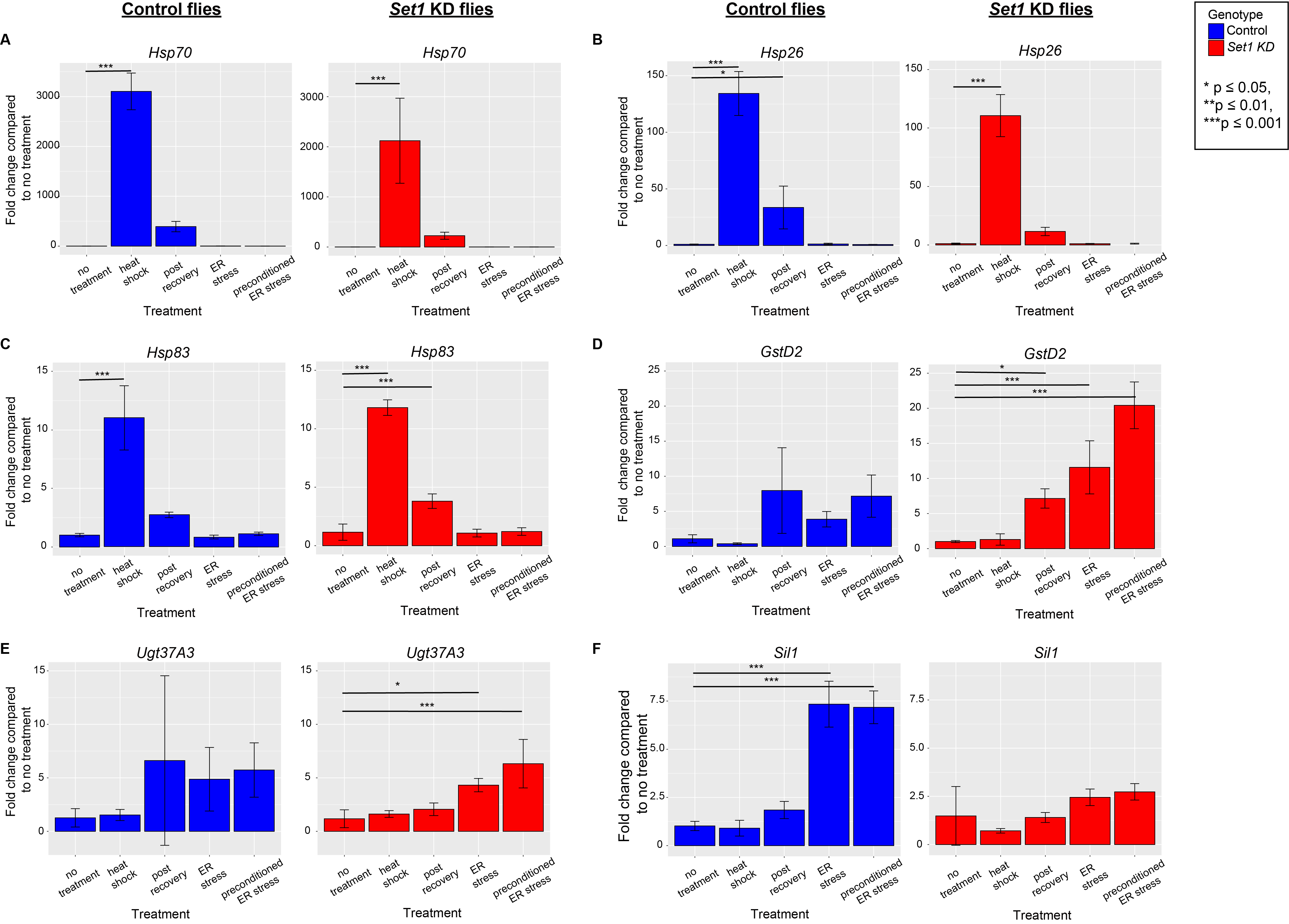

Supplement: jkad265_Supplementary_Data [file jkad265_supplementary_data.zip › Figure_S4_G3-2023-404642.tif]
